# Mitotic Nuclei Segmentation and Classification Using Chaotic Butterfly Optimization Algorithm with Deep Learning on Histopathology Images

**DOI:** 10.3390/biomimetics8060474

**Published:** 2023-10-05

**Authors:** Rayed AlGhamdi

**Affiliations:** Department of Information Technology, Faculty of Computing and Information Technology, King Abdulaziz University, Jeddah 21589, Saudi Arabia; raalghamdi8@kau.edu.sa

**Keywords:** deep learning, computer-aided diagnosis, mitotic nuclei classification, segmentation, metaheuristics

## Abstract

Histopathological grading of the tumors provides insights about the patient’s disease conditions, and it also helps in customizing the treatment plans. Mitotic nuclei classification involves the categorization and identification of nuclei in histopathological images based on whether they are undergoing the cell division (mitosis) process or not. This is an essential procedure in several research and medical contexts, especially in diagnosis and prognosis of cancer. Mitotic nuclei classification is a challenging task since the size of the nuclei is too small to observe, while the mitotic figures possess a different appearance as well. Automated calculation of mitotic nuclei is a stimulating one due to their great similarity to non-mitotic nuclei and their heteromorphic appearance. Both Computer Vision (CV) and Machine Learning (ML) approaches are used in the automated identification and the categorization of mitotic nuclei in histopathological images that endure the procedure of cell division (mitosis). With this background, the current research article introduces the mitotic nuclei segmentation and classification using the chaotic butterfly optimization algorithm with deep learning (MNSC-CBOADL) technique. The main objective of the MNSC-CBOADL technique is to perform automated segmentation and the classification of the mitotic nuclei. In the presented MNSC-CBOADL technique, the U-Net model is initially applied for the purpose of segmentation. Additionally, the MNSC-CBOADL technique applies the Xception model for feature vector generation. For the classification process, the MNSC-CBOADL technique employs the deep belief network (DBN) algorithm. In order to enhance the detection performance of the DBN approach, the CBOA is designed for the hyperparameter tuning model. The proposed MNSC-CBOADL system was validated through simulation using the benchmark database. The extensive results confirmed the superior performance of the proposed MNSC-CBOADL system in the classification of mitotic nuclei.

## 1. Introduction

The Breast Cancer (BC) mortality rate can be reduced only if the disease is diagnosed at early stages since its treatment strategy is directed according to the prognosis and grade of the tumor [1]. In order to categorize the severity of BC, the Nottingham Grading System (NGS) is extensively utilized. This system has three biomarkers to grade the BC using the histopathology images. The biomarkers are mitotic cell count, tubule formation, and nuclear atypia [2]. Among these three biomarkers, the number of mitotic cells is a significant biomarker as the mitotic cell division process is directly relevant to the diagnosis of cancer. In general, the mitotic cells can be identified by visually analyzing the breast histopathology images on higher-resolution microscopes [3]. However, this process is too subjective and hard and is a time-consuming one. A less experienced pathologist may incorrectly diagnose or stage the disease which, in turn, has a major impact on the patient’s life. Histopathological image analysis can be used for the detection of lung and colon cancer as well by investigating the microscopic images of the tissue samples [4]. Additionally, the existence of the mitotic nuclei in HPF differs based on the stages and grades of cancer [5]. In cancerous lesions, the mitotic nuclei look smaller in general with non-differentiable and maximum frequency. The diagnostic accuracy of the mitotic nuclei is based on the skills and proficiency of the pathologists.

The digitalization of histopathology technique and the developments in Machine Learning (ML) and medical image processing approaches paved the way for computer-aided pathology in recent years [6]. Accordingly, various automated systems were designed, for example, the automated classification and identification systems for nuclei, cancerous tissues, detection of biomarkers, and so on [7]. With the emergence of digital pathology systems, several computational techniques were presented for automated pathological outcomes. The latest developments in DCNNs and their excellent effectiveness in image classification, segmentation, and identification have increased their application in medical imaging devices [8]. A DCNN is a kind of representative learning method that can automatically extract the appropriate data from raw images without any need for manual development of the feature descriptors [9]. In the literature, the CNN-based methods were efficiently implemented to address numerous histopathological difficulties such as the diagnosis of cancer metastasis, demarcation of cancerous areas, determination of lymphocytes, classification of the breast tissue into benign, normal, invasive, and in situ carcinoma, segmentation of cell nuclei, and so on [10].

The current research article introduces the mitotic nuclei segmentation and classification using the chaotic butterfly optimization algorithm with deep learning (MNSC-CBOADL) technique on the histopathology images. The main objective of the MNSC-CBOADL system is to accomplish automated segmentation and classification of the mitotic nuclei. In the presented MNSC-CBOADL technique, the U-Net model is initially applied for the purpose of segmentation. Additionally, the MNSC-CBOADL technique applies the Xception model for feature vector generation. For the classification process, the MNSC-CBOADL technique employs the Deep Belief Network (DBN) approach. In order to enhance the detection performance of the DBN algorithm, the CBOA is designed for the hyperparameter tuning model. The proposed MNSC-CBOADL system was validated through simulation using the benchmark database. The extensive results established the superior performance of the MNSC-CBOADL algorithm in the classification of the mitotic nuclei. Some of the key contributions of the paper are summarized herewith.

- Development of an intelligent MNSC-CBOADL technique comprising pre-processing, U-Net segmentation, Xception-based feature extraction, DBN classification, and CBOA-based parameter tuning for mitosis cell nuclei segmentation and classification. To the best of the authors’ knowledge, the MNSC-CBOADL model has never been presented in the literature;
- U-Net segmentation is used to accurately delineate the mitotic nuclei from complex tissue images, while the DBN model can effectively model complex patterns in the data for classification;
- The CBOA algorithm is used for the optimization of the hyperparameters of the DBN model using cross-validation, which helps in boosting the predictive outcomes of the MNSC-CBOADL model for unseen data.

The rest of the paper is organized as follows: Section 2 provides the related works, and Section 3 discusses the proposed model. Then, Section 4 details the analytical outcomes, while Section 5 concludes the paper.

## 2. Related Works

In the study conducted earlier [11], the authors developed an automatic mitosis identification system from the histopathology images and grading method by employing the SVM method. Early diagnosis of cancer and prior knowledge about the patient’s medical history are crucial, and the proposed histopathological grading system for carcinoma was analyzed in this background. In the traditional approach, NGS was used for grading various stages of carcinoma. Khan et al. [12] introduced the SMDetector, a DL technique in which the dilated layers aim to reduce the size gap between the images and objects. Mathew et al. [13] suggested a novel method based on a class imbalance phenomenon which is understood by the growth of mitotic cells in a context-preserving way. Eventually, the adapted CNN algorithm was employed for the classification of the candidate cells into target class labels.

Shwetha and Dharmanna [14] presented a new CAD system with five phases. In the first phase, the images were pre-processed based on an image fine-tuning method. In the second phase, both the background and the foreground were segmented by following the Otsu segmentation approach. In the third phase, the bit plane slicing method was implemented to separate the non-mitotic and mitotic cells. In the fourth phase, the number of mitotic cells was calculated. At last, the phases of the cancer were diagnosed depending on the mitotic cell counts. Malibari et al. [15] developed the Artificial HBA with TL-based Mitotic Nuclei Classification (AHBATL-MNC) method using the histopathologic images of BC. In this histopathologic image segmentation method, the PSPNet technique was employed for analyzing the candidate mitotic regions. Later, the ResNet algorithm was used for feature extraction, and the XGBoost technique was implemented. In the study conducted earlier [16], a new architecture was introduced by employing NN-based approaches with fewer feature vectors and several ML methods. For this study, the authors implemented extraction with many approaches such as LTP, LBP, and GLCM, as well as classification methods, namely, RF, SVM, and NBs, for the source database of the images.

Sohail et al. [17] recommended an automated label refiner to characterize the weak labels using semi-sematic data for training the DCNNs. In this study, the authors utilized deep instance-based segmentation and identification techniques to explore the possible mitotic areas on tissue patches. Highly possible fields were screened based on the blob region, after which the cell level was identified by improving the conventional CNN method “MitosRes-CNN” so as to filter incorrect mitoses. Samah et al. [18] suggested a method to identify the mitotic cells from H- and E-stained overall-slide images of BC. This approach had a total of three phases, namely, the super-pixel segmentation for the collection of the same pixels into super-pixel areas, blob detection for the separation of the cells from the background and the tissues, and, finally, the classification and shape analysis. The suggested technique, along with a Fourier Descriptor (FD) and the Histogram of the Oriented Gradients (HOGs) as features, was applied to analyze the mitotic cells in a reliable manner. 

In the literature [19], the authors developed a novel partially supervised technique based on two parallel deep fully convolutional networks. Of these two, one was trained to employ the weak labels, whereas the other one was trained through strong labels, collected with a weight transfer function. During the identification stage, the authors combined the segmentation maps generated by both networks to accomplish the final mitotic analysis. Wahab et al. [20] developed a novel TL model by initially utilizing a pre-trained CNN for segmentation followed by another hybrid CNN and the weights transfer and custom layers for the classification of mitoses. Primarily, the mitotic nuclei are automatically annotated based on the ground truth centroids. The segmentation technique categorizes the mitotic nuclei, but it may also generate false positives at times. Mahmood et al. [21] introduced the multiphase mitotic cell identification technique derived from deep CNNs and a Faster Region-CNN (Faster R-CNN).

## 3. The Proposed Model

**Figure 1:**
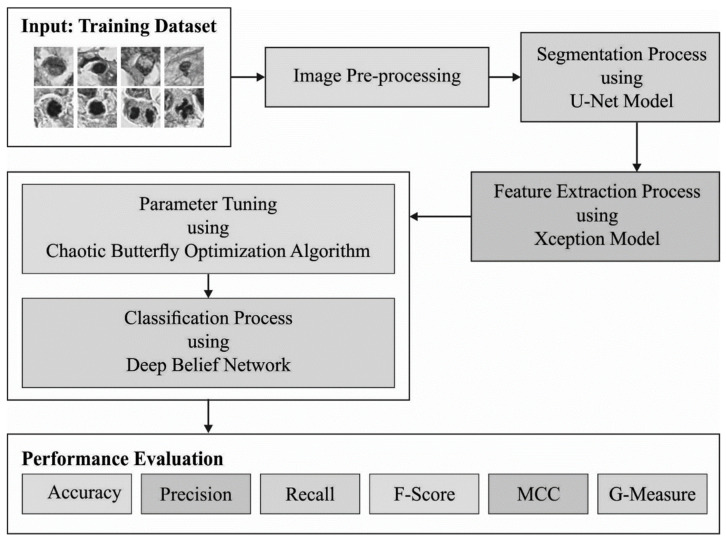
Workflow of the MNSC-CBOADL algorithm.

In the current study, the automated MNSC-CBOADL technique is proposed, designed, and validated for its performance in terms of automated mitotic nuclei segmentation and classification upon the histopathology images. The key objective of the MNSC-CBOADL system is to accomplish automated segmentation and classification of the mitotic nuclei. In the presented MNSC-CBOADL technique, different stages of operations are involved, namely, U-Net segmentation, Xception feature extraction, DBN classification, and CBOA-based hyperparameter tuning. Figure 1 depicts the workflow of the MNSC-CBOADL system.

### 3.1. Image Segmentation

For the segmentation process, the U-Net model is applied. The U-Net structure has two major paths [22], the contraction path and expansion path. The contraction path is called the encoder, which is accountable for capturing the image context using max-pooling and convolutional layers. On the other hand, the expansion path is called the decoder, which is accountable for localization and object detection using the transposed convolution. Generally, the encoded path reduces the spatial resolution of the input images, while, with the help of an up-sampling layer, the decoder gradually recovers the spatial resolution. The U-Net structure can handle images of any size without dense layers. The skip connection is utilized for connecting the encoder block output to its respective decoder block. This stage tries to recover the fine details that are learnt through encoding to restore the spatial resolution of the novel input images.

The contracting path implements a down-sampling process that comprises two 3 × 3 convolution layers, followed by 2 × 2 max-pooling with stride 2 and the ReLU activation function. The feature channel counts are improved by a factor of 2 for every down-sampling, whereas the expansive path implements the up-sampling process. It has a 2 × 2 convolution layer that decreases the number of feature channels by half followed by a concatenation with respective features in the contracting path and two 3 × 3 convolutional layers and, finally, the ReLu function. Finally, a 1 × 1 convolutional layer is used for mapping the 64-element feature vectors to the required number of classes. The convolution can be obtained using Equation (1), which is implemented as a kind of transformation.

(1) $$Z\left(x_{k}\left(ii,jj\right)\right)=f\left(\sum_{k=1}^{k}x_{k}\left(ii,jj\right)\cdot w_{k}+b_{k}\right)↔Z=f\left(X.W+b\right),$$

In Equation (1), w represents the weight vector, b corresponds to the bias vector, and $x_{k}$ (ii,jj) refers to the input of the activation function and output of the convolution operation. After the convolution process becomes completed, the U-Net structure uses ReLU as an activation function as follows:

(2) $$A\left(x_{k}\left(ii,jj\right)\right)=max(0,Z\left(x_{k}\left(ii,jj\right)\right).$$

### 3.2. Feature Extraction

In this stage, the Xception method is utilized to derive the feature vectors. The Xception structure called ‘Extreme Inception’ is a CNN structure that comprises a series of depthwise separable convolutional layers with remaining connections [23]. This structure contains 36 convolution layers that are collected as 14 blocks, where the first and the last blocks feature the linear residual connections amongst the others. In order to enhance the accuracy of the model and extract high-level features from the histopathological images, custom layers in the procedure of three convolutions and three max-pooling layers are used together with the pre-training structure. The Xception method weights can initialize the ImageNet weights. The flattening function changes the mapping feature achieved earlier to a 1D vector. Here, dropout is employed to reduce the overfitting issues, whereas batch normalization is employed as a regularized system. The last sigmoid activation function provides the outcome for class probability in the range of 0 to 1. Afterwards, the entire CNN structure containing the pre-training method (FC layers) and custom layers is trained using the augmented BreakHis database with a 40× magnification aspect.

### 3.3. Classification Using the DBN Model

For the classification process, the DBN approach is followed. DBN is a multi-layer NN architecture and is a multi-layer probabilistic ML algorithm [24]. The conventional MLP technique is confronted with a few complications such as gradient vanishing, time-consuming processes, and a huge demand for training datasets; nevertheless, the DBN is an advanced DL technique that can overcome these drawbacks. The DBN has both unsupervised and supervised learning techniques, where the former can be obtained by the network architecture with a multi-layer of RBM bodies. On the other hand, the backpropagation network layer implements supervised learning. Unsupervised learning finalizes the initialization parameter of all the layers of network architecture, whereas the supervised learning process fine-tunes the initial parameters globally. 

RBM consists of a visible layer (VL) and hidden layers (HL). The HL and VL are interconnected in both the directions, whereas the nodes of all the layers are not interconnected with one another. In the RBM learning method, the $E\left(v,h|\theta \right)$ energy function is determined as follows:

(3) $$E\left(v,h|\theta \right)=-\sum_{i=1}^{n}\sum_{j=1}^{m}\omega_{ij}v_{i}h_{j}-\sum_{i=1}^{n}b_{i}v_{i}-\sum_{j=1}^{m}c_{j}h_{j}$$

**Figure 2:**
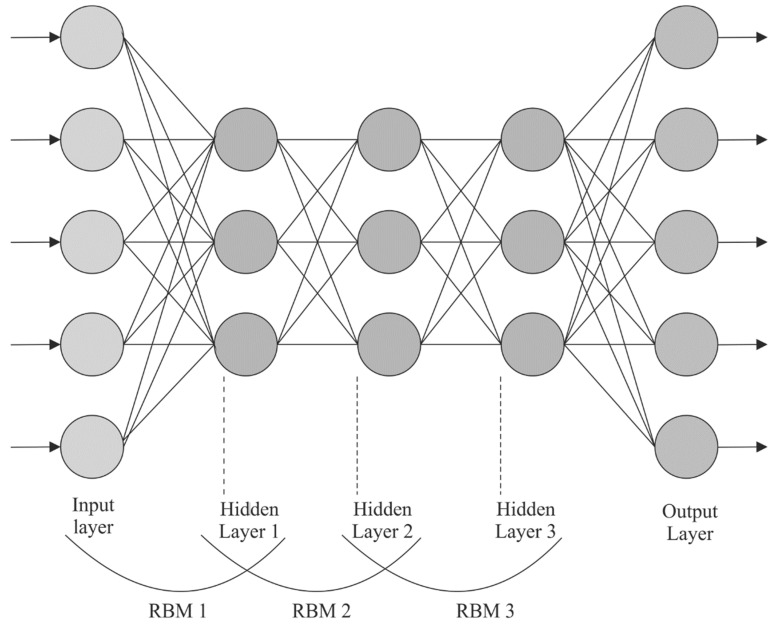
DBN structure.

In Equation (3),$\;\theta =\{\omega_{ij},{\;b}_{i},c_{j}\}$ corresponds to the parameter set of RBM. $v=(v_{1},v_{2},\cdots ,v_{n}{)}^{T}$ represents the VL, $h=(h_{1},h_{2},\cdots ,{\;h}_{m})$ indicates the HL, $\omega =(\omega_{i,j})\in R^{n\times m}$ shows the weight matrix that interconnects both the layers, and $b=(b_{1},b_{2},\cdots ,{\;b}_{n}{)}^{T}$ and $c=(c_{1},c_{2},\cdots ,c_{m}{)}^{T}$ refers to the bias of v and h, correspondingly. Figure 2 depicts the framework of the DBN.

This RBM architecture enables the VL and HL values to be unrelated to one another. The whole layer is computed in parallel instead of calculating all the neurons. Next, the probability distribution of VL and HL is given below:

(4) $$p\left(v,h|\theta \right)=\frac{e^{-E\left(v,h|\theta \right)}}{Z\left(\theta \right)}\;$$

(5) $$Z\left(\theta \right)=\sum_{v}\sum_{h}e^{-E\left(v,h|\theta \right)}\;$$

Here, Z(θ) refers to the normalized constant.

Therefore, the neuron probability $h_{j}$ is activated in the HL of RBM as follows:

(6) $$p\left(h_{j}=1|v;\theta \right)=f\left(c_{j}+\sum_{i=1}^{n}\omega_{ij}v_{i}\right)\;$$

Meanwhile, the RBM layer is interconnected in two directions. The neurons in the visible layer $v_{i}$ are activated by the neuron $h_{j}$ in HL, and its probability is formulated using Equation (7):

(7) $$p\left(v_{i}=1|h;\theta \right)=f\left(b_{i}+\sum_{j=1}^{m}\omega_{ij}h_{j}\right)\;$$

The RBM training method learns the values of θ parameters to fit into the training dataset. Usually, the non-supervised RBM learning method exploits the Contrastive Divergence (CD) method to update the parameters, and the updating rules for all the parameters are given below:

(8) $$\Delta \omega =\varepsilon \left(E_{data}\left(v_{i}h_{j}\right)-E_{recon}\left(v_{i}h_{j}\right)\right)$$

(9) $$\Delta c=\varepsilon \left(E_{data}\left(h_{j}\right)-E_{recon}\left(h_{j}\right)\right)$$

(10) $$\Delta b=\varepsilon \left(E_{data}\left(v_{i}\right)-E_{recon}\left(v_{i}\right)\right)$$

Here, $E_{econ}$ indicates the expectation over distribution described by the reconstruction mechanism, $E_{data}$ denotes the mathematical expectation with distribution described by the trained data, and ε refers to the learning rate for training the RBM.

### 3.4. Hyperparameter Tuning Using CBOA

Finally, the CBOA is utilized for the selection of the optimum hyperparameters of the DBN algorithm. The BOA is a swarm optimization approach inspired by the natural behavior of social butterflies during foraging [25]. The BOA searches globally as well as locally for a better solution. In the current research work, the data are transmitted to other solutions (searching agents) using smell to form the combined social networks. Naturally, butterflies use a sensor to smell or sense fragrance. According to their fitness, all the butterflies scatter a dissimilar amount of fragrance. A butterfly discharges a strong concentrated smell when it moves.

(11) $$pf_{i}=cI^{a}\;$$

In Equation (11), $pf_{i}$ corresponds to the perceived magnitude of fragrance, while a and c are the parameters that correspond to sensing modality and the power exponent, correspondingly. I denotes the stimulus concentration.

A parameter is a power exponent that defines the dissimilarity of odor absorption, thus affecting the butterfly’s capability to search for a better outcome. For a=1, there is no absorption of fragrance. Other butterflies sense each amount of the fragrance released by the butterfly particles. For a=0, the fragrance released by the butterfly particles is not perceptible to the rest of the butterfly individuals.

(12) $$a\left(t\right)=a_{s}-\left(a_{s}-a_{f}\right)\times sin\left(\left(\frac{\pi }{\mu }\right)\times \left(\frac{t}{T_{max}}\right)2\right)$$

In Equation (12), $a_{s}$ and $a_{f}$ indicate the first and last values of a, μ shows the parameter tuning, and $T_{max}$ represents the maximum iteration counter. The value of sensor modality c lies in the range of 0 to 1. The value is updated in an iterative manner using Equation (13):

(13) $$c_{t+1}=c_{t}+\left(\frac{0.025}{c_{t}\times T_{max}}\right)$$

Here, $T_{max}$ denotes the maximal iteration counter, and the initial value of c is 0.01.

All the butterflies emit fragrance once they move, and the rest of the butterflies are attracted to it based on their amount of fragrance. This phenomenon is named ‘global search’ and is determined using Equation (14):

(14) $$x_{i}^{t+1}=x_{i}^{t}+\left(r^{2}xg*-x_{i}^{t}\right)\times f_{i}$$

In Equation (14), $x_{i}^{t}$ refers to the vector that signifies the solution (butterfly) at the $t^{th}$ iteration, g* indicates the overall better solution, r shows the randomly generated value within [0,1], and $f_{i}$ indicates the fragrance of the $i^{th}$ butterfly. Once a butterfly fails to smell the odor concentration of others, it randomly moves into the search region. This phenomenon is named the ‘local search’ process and is determined using Equation (15):

(15) $$x_{i}^{t+1}=x_{i}^{t}+\left(r^{2}\times x_{j}^{t}-x_{k}^{t}\right)\times f_{i}$$

Here, $x_{j}^{t}$ and $x_{k}^{t}$ correspond to two vectors that signify two dissimilar butterflies in a similar population.

### 3.5. Chaotic Butterfly Optimization Algorithm (CBOA)

The CBOA is a revised version of the BOA that exploits chaotic maps instead of the randomly generated parameters in Equations (15) and (16) for updating the position of the butterfly. This phenomenon improves the performance of the BOA.

(16) $$x_{i}^{t+1}=x_{i}^{t}+\left(C^{2}\times g*-x_{i}^{t}\right)\times f_{i}$$

In Equation (16), $x_{i}^{t}$ indicates the vector that shows the butterfly (solution) at the t iteration, g* represents the overall better performance, C implies the chaotic value, and $f_{i}$ shows the fragrance of the $i^{th}\;$butterfly.

(17) $$x_{i}^{t+1}=x_{i}^{t}+\left(C^{2}\times x_{j}^{t}-x_{k}^{t}\right)\times f_{i}$$

In Equation (17), two vectors, $x_{j}^{t}$ and $x_{k}^{t}$, indicate different butterflies in a similar population.

The CBOA system grows a Fitness Function (FF) to accomplish excellent classification outcomes. It explains a positive integer to depict the good solution for candidate performances. In the current study, the reduction in classifier errors is assumed to be an FF, as provided in Equation (18):

(18) $$fitness\left(x_{i}\right)=ClassifierErrorRate\left(x_{i}\right)\;=\frac{No.\;of\;misclassified\;instances\;}{Total\;no.\;of\;instances}\times 100$$

## 4. Results and Discussion

The proposed model was simulated using the Python 3.6.5 tool configured on a PC with specifications such as i5-8600k, GeForce 1050Ti 4GB, 16GB RAM, 250GB SSD, and 1TB HDD. The classification performance of the MNSC-CBOADL system was validated utilizing a standard database [26], comprising 150 samples, as depicted in Table 1. The dataset comprises images in the form of whole-slide images, and they are saved in Aperio .svs file format as multi-resolution pyramid structures (the size of the highest resolution image can easily exceed 50,000 by 50,000 pixels). Every image in the pyramid is saved as a series of tiles in order to facilitate the rapid retrieval of subregions in the image. Each case is represented with a single whole-slide image and is annotated with a proliferation score based on mitotic counting by pathologists and molecular proliferation score.

**Figure 3:**
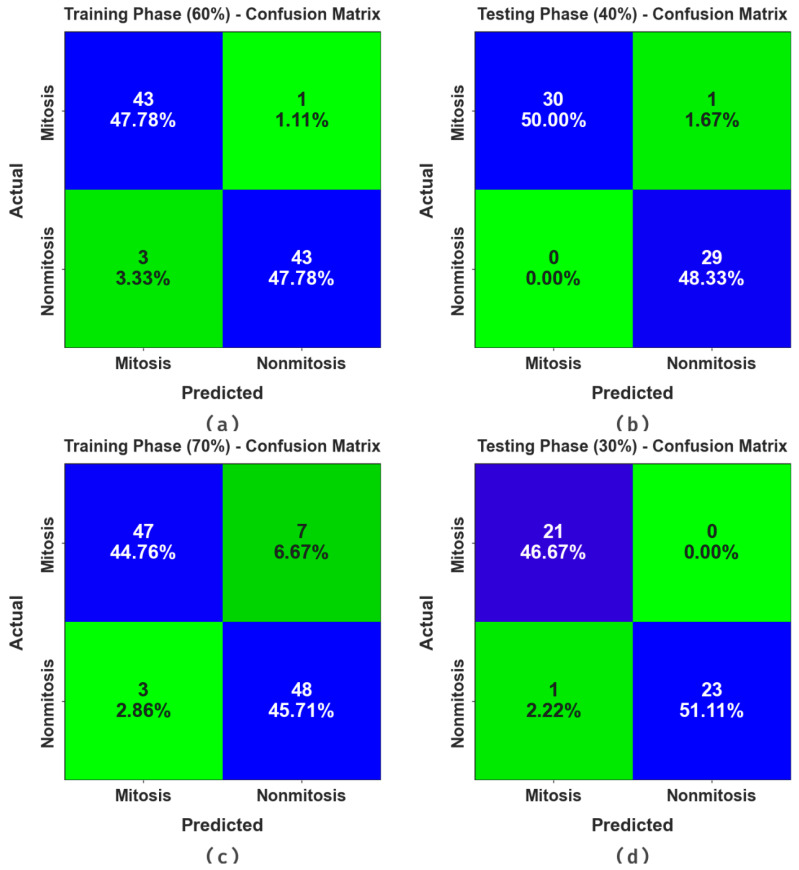
Confusion matrices of (**a**,**b**) 60:40 of TR set/TS set and (**c**,**d**) 70:30 of TR set/TS set.

Figure 3 portrays the confusion matrices generated by the MNSC-CBOADL system for distinct databases. The simulation value shows that the MNSC-CBOADL methodology detected and classified the mitotic and non-mitotic classes accurately.

**Figure 4:**
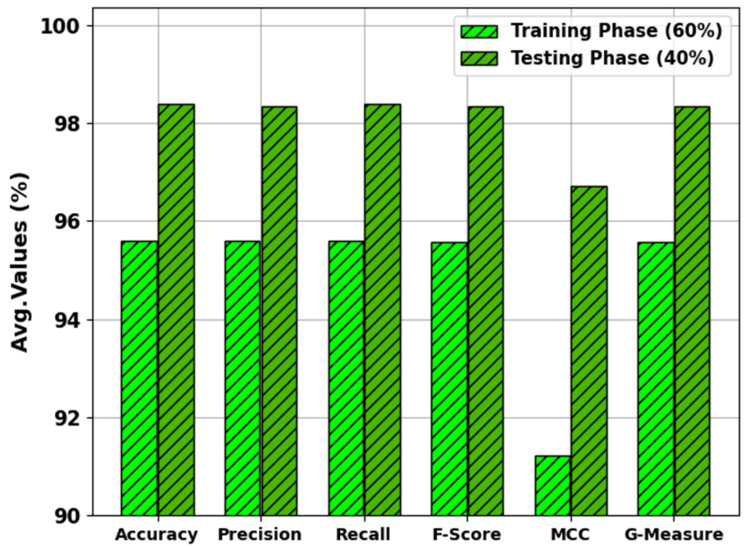
Average values of the MNSC-CBOADL algorithm at 60:40 of TR set/TS set.

In Table 2 and Figure 4, the results of the MNSC-CBOADL approach under 60:40 of the TR set/TS set are shown. The MNSC-CBOADL technique properly recognized the mitotic and non-mitotic class samples. With the 60% TR set, the MNSC-CBOADL technique attained an average $accu_{y}$ of 95.60%, $prec_{n}$ of 95.60%, $reca_{l}$ of 95.60%, $F_{score}$ of 95.56%, MCC of 91.21%, and a $G_{measure}$ of 95.58%. In addition, on the 40% TS set, the MNSC-CBOADL approach achieved an average $accu_{y}$ of 98.39%, $prec_{n}$ of 98.33%, $reca_{l}$ of 98.39%, $F_{score}$ of 98.33%, MCC of 96.72%, and a $G_{measure}$ of 98.35%.

**Figure 5:**
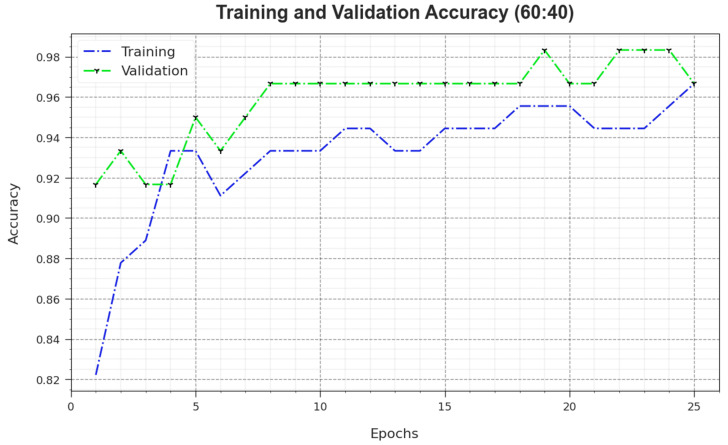
$Accu_{y}$ curve of the MNSC-CBOADL algorithm at 60:40 of TR set/TS set.

Figure 5 illustrates the training accuracy $TR\_accu_{y}$ and $VL\_accu_{y}$ values achieved by the MNSC-CBOADL algorithm upon the 60:40 TR set/TS set. The $TL\_accu_{y}$ is defined as an estimate of the MNSC-CBOADL system for the TR dataset, whereas the $VL\_accu_{y}$ value is computed by evaluating the performance of the model upon a separate testing dataset. The outcomes display that $TR\_accu_{y}$ and $VL\_accu_{y}$ values upsurge with an increase in the number of epochs. Accordingly, the performance of the MNSC-CBOADL method improved on TR and TS datasets, with an increase in the number of epochs.

**Figure 6:**
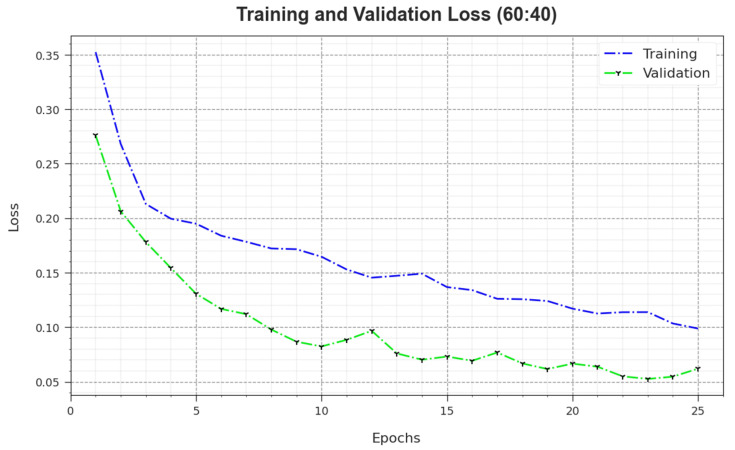
Loss curve of the MNSC-CBOADL algorithm at 60:40 of TR set/TS set.

In Figure 6, the TR_loss and VR_loss outcomes of the MNSC-CBOADL algorithm on the 60:40 TR set/TS set are exposed. The TR_loss value demonstrates the error between the predictive outcome and original values of the TR data. The VR_loss value measures the performance of the MNSC-CBOADL algorithm on individual validation data. These results indicate that TR_loss and VR_loss values tend to be lesser with an increase in the number of epochs. It portrays the enhanced outcomes of the MNSC-CBOADL method and its ability to generate accurate classification. The minimal TR_loss and VR_loss values establish the enhanced performance of the MNSC-CBOADL approach in terms of capturing the patterns and relationships.

**Figure 7:**
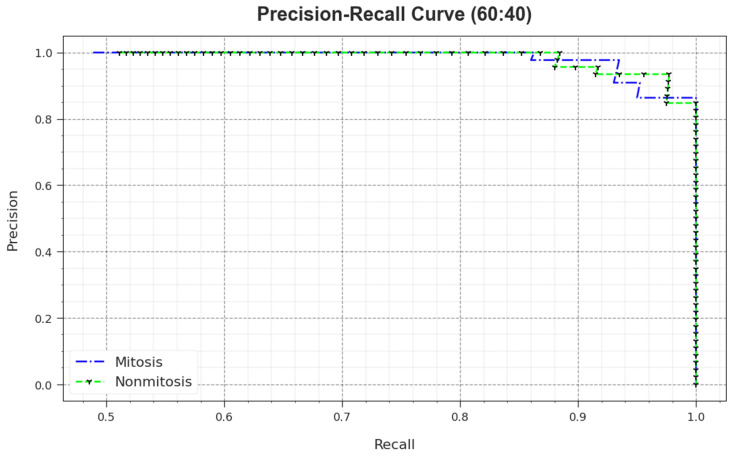
PR curve of the MNSC-CBOADL algorithm on 60:40 of TR set/TS set.

A detailed PR outcome of the MNSC-CBOADL algorithm for 60:40 of the TR set/TS set is shown in Figure 7. The simulation outcomes demonstrate that the MNSC-CBOADL approach achieved enhanced PR values. Moreover, the MNSC-CBOADL algorithm attained superior PR performances on two classes.

**Figure 8:**
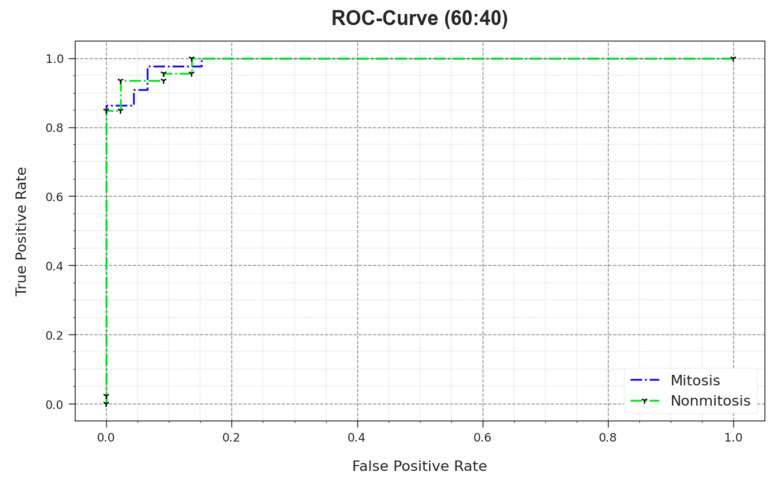
ROC of the MNSC-CBOADL algorithm on 60:40 of TR set/TS set.

In Figure 8, the ROC outcomes of the MNSC-CBOADL method are demonstrated on the 60:40 TR set/TS set. The outcomes show that the MNSC-CBOADL system increased the ROC values. Thus, it is obvious that the MNSC-CBOADL algorithm achieved superior ROC performance on both the classes.

**Figure 9:**
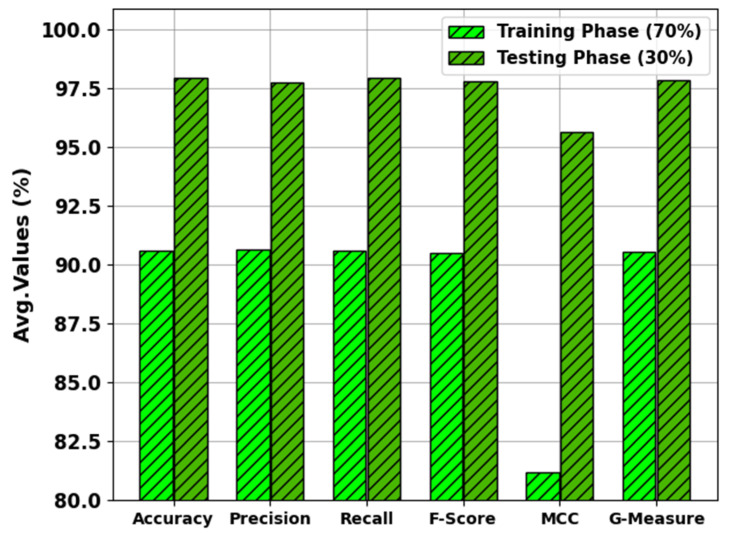
Average values of the MNSC-CBOADL algorithm at 70:30 of TR set/TS set.

In Table 3 and Figure 9, the results of the MNSC-CBOADL approach on 70:30 of the TR set/TS set are portrayed. The MNSC-CBOADL algorithm properly identified both mitotic and non-mitotic class samples. On the 70% TR set, the MNSC-CBOADL algorithm achieved an average $accu_{y}$ of 90.58%, $prec_{n}$ of 90.64%, $reca_{l}$ of 90.58%, $F_{score}$ of 90.48%, MCC of 81.21%, and a $G_{measure}$ of 90.54%. Afterwards, on the 30% TS set, the MNSC-CBOADL methodology accomplished an average $accu_{y}$ of 97.92%, $prec_{n}$ of 97.73%, $reca_{l}$ of 97.92%, $F_{score}$ of 97.77%, MCC of 95.64%, and a $G_{measure}$ of 97.80%.

**Figure 10:**
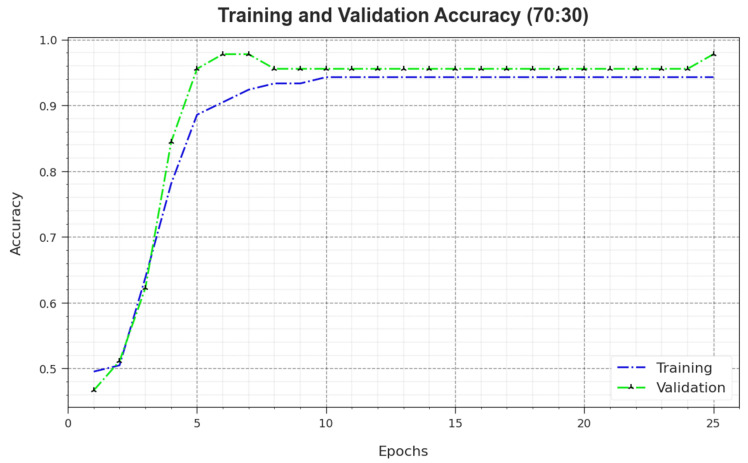
$Accu_{y}$ curve of the MNSC-CBOADL algorithm at 70:30 of TR set/TS set.

Figure 10 depicts the training accuracy $TR\_accu_{y}$ and $VL\_accu_{y}$ values of the MNSC-CBOADL algorithm for the 70:30 TR set/TS set. $TL\_accu_{y}$ is defined as an estimation of the MNSC-CBOADL algorithm on the TR dataset, whereas the $VL\_accu_{y}$ value is calculated by assessing the performance on a separate testing dataset. The outcomes exhibit that both $TR\_accu_{y}$ and $VL\_accu_{y}$ values increase with an upsurge in the number of epochs. So, the outcome of the MNSC-CBOADL technique was improved on both TR and TS datasets with an increase in the number of epochs.

**Figure 11:**
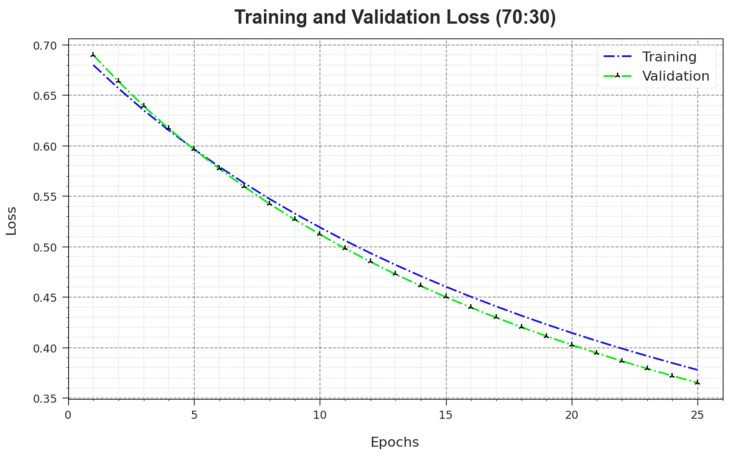
Loss curve of the MNSC-CBOADL algorithm at 70:30 of TR set/TS set.

In Figure 11, the TR_loss and VR_loss results of the MNSC-CBOADL approach on the 70:30 TR set/TS set are revealed. TR_loss defines the error between the predictive solution and original values on the TR data. VR_loss signifies the performance measure of the MNSC-CBOADL technique on individual validation data. The results indicate that both TR_loss and VR_loss values tend to reduce with rising epochs. This phenomenon describes the greater solution of the MNSC-CBOADL technique and its ability to generate accurate classification. The low TR_loss and VR_loss values reveal the enhanced outcome of the MNSC-CBOADL method in terms of capturing the patterns and relationships.

**Figure 12:**
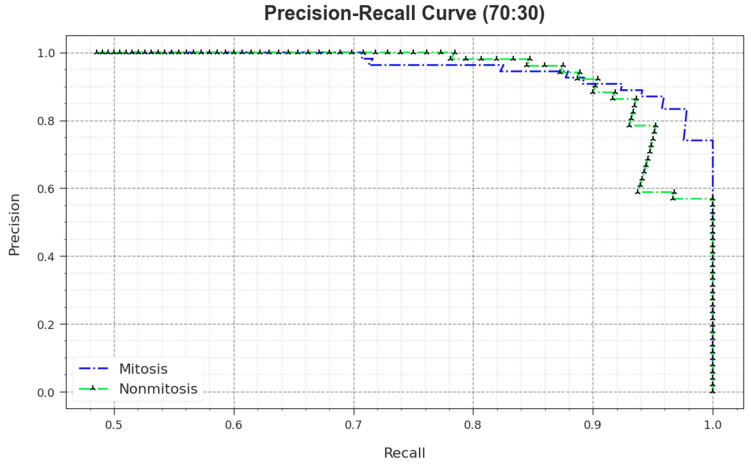
PR curve of the MNSC-CBOADL algorithm at 70:30 of TR set/TS set.

A comprehensive PR study was conducted upon the MNSC-CBOADL method using the 70:30 TR set/TS set, and the outcomes are shown in Figure 12. The simulation values denote that the MNSC-CBOADL system achieved superior PR outcomes. Thus, it is evident that the MNSC-CBOADL approach gained better PR values on both the classes.

**Figure 13:**
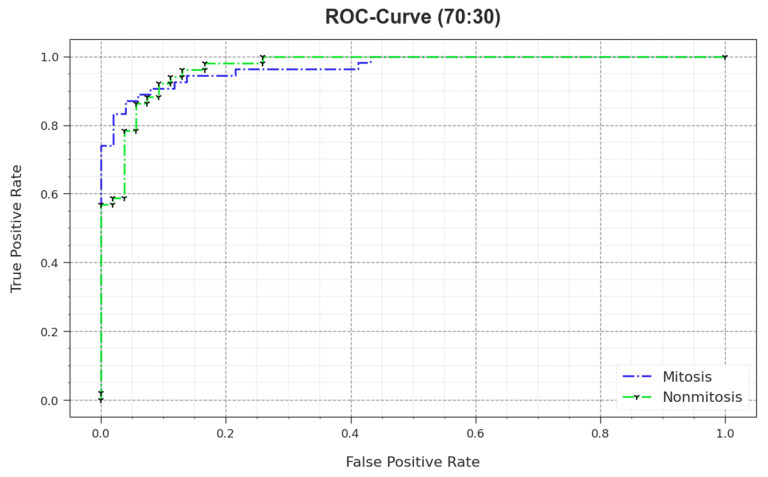
ROC value of the MNSC-CBOADL algorithm on 70:30 of TR set/TS set.

In Figure 13, the ROC curve is shown for the MNSC-CBOADL system upon the 70:30 TR set/TS set. The results demonstrate that the MNSC-CBOADL technique produced the optimum ROC values. So, the MNSC-CBOADL approach attained excellent performances in terms of ROC on both the classes.

**Figure 14:**
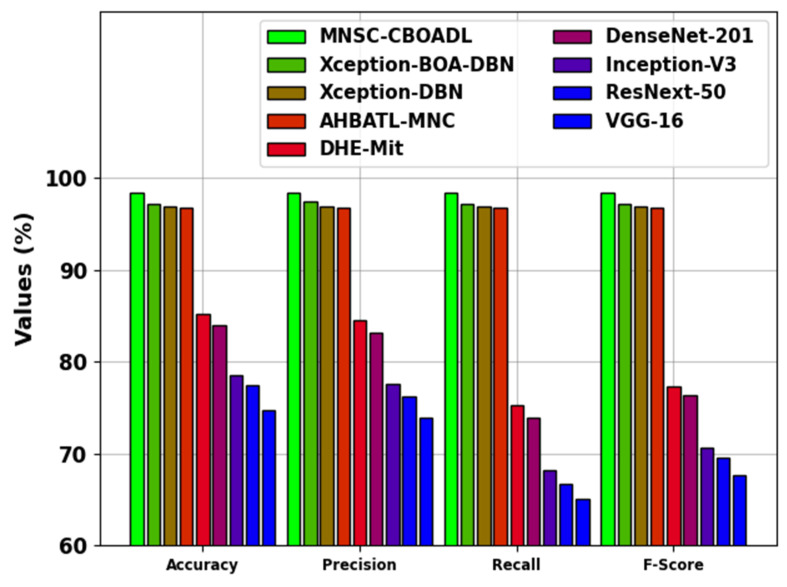
Comparative outcome of MNSC-CBOADL algorithm with existing methods.

The comparative analysis results of the MNSC-CBOADL technique are depicted in Table 4 and Figure 14 [15,27]. The outcomes show that the MNSC-CBOADL technique achieved promising results over other models. In terms of $accu_{y}$, the MNSC-CBOADL technique achieved a maximum $accu_{y}$ of 98.39%. At the same time, the AHBATL-MNC, DHE-Mit, DenseNet201, Inception-V3, ResNext-50, and VGG-16 models reached low $accu_{y}$ values of 96.77%, 85.23%, 83.96%, 78.54%, 77.48%, and 74.72%, respectively. It was also noticed that the Xception-BOA-DBN and Xception-DBN models managed to achieve a considerable performance. However, the proposed model achieved a better performance over other models under different measures. The enhanced performance of the proposed model is due to the inclusion of the CBOA-based hyperparameter tuning process.

The above-discussed performances established the highest classification efficiency of the proposed MNSC-CBOADL methodology.

## 5. Conclusions

In the current study, an automated MNSC-CBOADL technique was designed and developed for automated mitotic nuclei segmentation and classification in histopathology images. The main purpose of the MNSC-CBOADL algorithm is to accomplish the automated segmentation and classification of mitotic nuclei. In the presented MNSC-CBOADL technique, different stages of operations are involved, namely, U-Net segmentation, Xception feature extraction, DBN classification, and CBOA-based hyperparameter tuning. For the classification process, the MNSC-CBOADL technique employs the DBN approach. In order to improve the detection performance of the DBN algorithm, the CBOA is designed for the hyperparameter tuning process. The proposed MNSC-CBOADL system was experimentally validated through simulation using the benchmark database. The extensive results confirmed the superior performance of the MNSC-CBOADL algorithm in terms of classification of mitotic nuclei, with a maximum accuracy of 98.39%. Thus, the proposed model can be employed for an automated and accurate mitotic nuclei classification process. In the future, the information from different imaging modalities such as brightfield and fluorescence microscopy can be included to improve the mitosis detection accuracy. The integration of data from various modalities can offer enriched contextual data and enhance the accuracy of the outcomes. In addition, the future works can also focus on the design of ensemble models that combine the predictions from multiple classifiers or neural networks to improve the mitotic classification accuracy.

## Figures and Tables

**Table 1:** Description of the database.

Class	No. of Samples
Mitosis	75
Nonmitosis	75
Total Samples	150

**Table 2:** Classifier outcomes of the MNSC-CBOADL algorithm at 60:40 of TR set/TS set.

Class	$Accu_{y}$	$Prec_{n}$	$Reca_{l}$	$F_{Score}$	MCC	$G_{Measure}$
**TR set (60%)**						
Mitosis	97.73	93.48	97.73	95.56	91.21	95.58
Nonmitosis	93.48	97.73	93.48	95.56	91.21	95.58
**Average**	**95.60**	**95.60**	**95.60**	**95.56**	**91.21**	**95.58**
**TS set (40%)**						
Mitosis	96.77	100.00	96.77	98.36	96.72	98.37
Nonmitosis	100.00	96.67	100.00	98.31	96.72	98.32
**Average**	**98.39**	**98.33**	**98.39**	**98.33**	**96.72**	**98.35**

**Table 3:** Classifier outcomes of the MNSC-CBOADL algorithm at 70:30 of TR set/TS set.

Class	$Accu_{y}$	$Prec_{n}$	$Reca_{l}$	$F_{Score}$	MCC	$G_{Measure}$
**TR set (70%)**						
Mitosis	87.04	94.00	87.04	90.38	81.21	90.45
Nonmitosis	94.12	87.27	94.12	90.57	81.21	90.63
**Average**	**90.58**	**90.64**	**90.58**	**90.48**	**81.21**	**90.54**
**TS set (30%)**						
Mitosis	100.00	95.45	100.00	97.67	95.64	97.70
Nonmitosis	95.83	100.00	95.83	97.87	95.64	97.89
**Average**	**97.92**	**97.73**	**97.92**	**97.77**	**95.64**	**97.80**

**Table 4:** Comparative outcomes of the MNSC-CBOADL algorithm and other existing methodologies [15,27].

Methods	$Accu_{y}$	$Prec_{n}$	$Reca_{l}$	$F_{Score}$
MNSC-CBOADL	98.39	98.33	98.39	98.33
Xception-BOA-DBN	97.12	97.47	97.08	97.16
Xception-DBN	96.89	96.91	96.85	96.86
AHBATL-MNC	96.77	96.77	96.77	96.67
DHE-Mit	85.23	84.45	75.26	77.33
DenseNet-201	83.96	83.20	73.85	76.38
Inception-V3	78.54	77.51	68.18	70.64
ResNext-50	77.48	76.20	66.73	69.49
VGG-16	74.72	73.93	65.00	67.66

## Data Availability

Data sharing does not apply to this article as no datasets were generated during the current study.
